# Medical theses as part of the scientific training in basic medical and dental education: experiences from Finland

**DOI:** 10.1186/1472-6920-7-51

**Published:** 2007-12-05

**Authors:** Pentti Nieminen, Kirsi Sipilä, Hanna-Mari Takkinen, Marjo Renko, Leila Risteli

**Affiliations:** 1 Medical Informatics Group, University of Oulu, P.O. Box 5000, FIN-90014 Oulu, Finland; 2 Institute of Dentistry, University of Oulu, P.O. Box 5000, FIN-90014 Oulu, Finland; 3 Oral and Maxillofacial Department, Oulu University Hospital, Aapistie 3, FIN-90220 Oulu, Finland; 4 Department of Paediatrics, University of Oulu, P.O. Box 5000, FIN-90014 Oulu, Finland; 5 Research and Innovation Services, University of Oulu, P.O. Box 8000, FIN-90014 Oulu, Finland

## Abstract

**Background:**

Teaching the principles of scientific research in a comprehensive way is important at medical and dental schools. In many countries medical and dental training is not complete until the candidate has presented a diploma thesis. The objective of this study was to evaluate the nature, quality, publication pattern and visibility of Finnish medical diploma theses.

**Methods:**

A total of 256 diploma theses presented at the University of Oulu from 2001 to 2003 were analysed. Using a standardised questionnaire, we extracted several characteristics from each thesis. We used the name of the student to assess whether the thesis resulted in a scientific publication indexed in medical article databases. The number of citations received by each published thesis was also recorded.

**Results:**

A high proportion of the theses (69.5%) were essentially statistical in character, often combined with an extensive literature review or the development of a laboratory method. Most of them were supervised by clinical departments (55.9%). Only 61 theses (23.8%) had been published in indexed scientific journals. Theses in the fields of biomedicine and diagnostics were published in more widely cited journals. The median number of citations received per year was 2.7 and the range from 0 to 14.7.

**Conclusion:**

The theses were seldom written according to the principles of scientific communication and the proportion of actually published was small. The visibility of these theses and their dissemination to the scientific community should be improved.

## Background

The teaching of scientific communication is an important part of basic medical education. Producing a thesis is an essential step for a student graduating from medical school [1]. It is also important that medical and dental undergraduate curricula should include the teaching of scientific thinking and the principles of scientific research [2]. In Finland, 10–20 weeks are reserved for advanced special studies in the medical and dental undergraduate curricula. These studies are compulsory and include an independent research project and the writing of a short diploma thesis [3]. Students are encouraged to consider research projects of any kind and mostly select their topic from ongoing scientific research projects in the university's departments and clinics. The academic staff at the departments act as supervisors for the theses.

Interest in the literature on this subject has focused on the role of writing a thesis as a part of medical education [1,4-6]. Teaching students to write effectively has been a major concern in education for many years [7,8]. Problems affecting such scientific writing have included variety in terms of scientific level and the requirements of the research projects as well as inadequate supervision. Problems have also arisen in the timing of the work, in that the courses that support scientific writing have been scheduled separately from the actual scientific work [9]. Hren et al. note, however, that medical students generally have a positive attitude towards science and scientific research [2]. Medical research and projects also have several benefits, such as improving students' ability to interpret the scientific literature critically when working as physicians or dentists, increasing the potential number of scientists who will pursue medical research and improving independent analytical problem-solving skills [6,10].

One indicator of the scientific value of a thesis and of the acceptability of its content to the international scientific community is whether the work has been published in a peer-reviewed journal. The quality of the theses can be judged by the proportion of published papers [5]. These peer-reviewed articles also give positive information about the integration of teaching and research within medical and dental education.

Our aim was to evaluate the graduate theses completed at a Finnish university and to examine their publication pattern and utility. Special attention was focused on supervision, the nature and quality of the theses and whether they were later published in the scientific literature.

## Methods

Our research material consisted of 256 consecutive medical and dental theses completed at the Medical Faculty of the University of Oulu in 2001–2003.

We reviewed the printed copy of each thesis supplied by the student to the faculty and evaluated several characteristics including language, department, supervisors and examiners. The theses were classified as 1) extensive analyses of quantitative material, 2) brief quantitative analyses of the authors' own material linked with a literature review or the development of a laboratory method, 3) pure literature reviews or 4) others (case reports, experimental generation of a laboratory procedures, analyses of qualitative material, descriptions of health organisations, or combinations of these).

Using a protocol for data collection, the following information was also obtained: does the report follow the structure of a scientific article (no, yes or printed publication), and what is its technical quality (unsatisfactory/satisfactory, good or excellent)? The shortcomings in technical quality were related to elementary word processing techniques and included the following: (i) disturbing variations in font, font style and font size, (ii) indefinite or inconsistent margins, (iii) inconsistent line spacing, (iv) unfinished tables and figures, (v) unfinished table of contents, or (vi) deficiencies in reference format, and (vii) others. The technical quality was evaluated as poor (several shortcomings), good (no more than two shortcomings) or excellent (no shortcomings). The technical quality of the printed publications was not evaluated.

The theses were categorized as using statistical methods if descriptive statistics (distributions, means, medians, standard deviations, etc.) or the results of formal tests of statistical significance were reported. The usage of tables and figures was also assessed. The presence or absence of special advanced statistical procedures and techniques was reviewed in each thesis. The same classification was used as in the study by Miettunen *et al.*[11]. Multivariate methods and survival time analyses, for example, were considered to be special methods. To evaluate the quality of the statistical reporting information was obtained on whether the data analysis procedures were completely described in the methods section. The description of methods was considered adequate if it satisfactorily explained what approaches and methods had been used to answer the main question posed in the research and why these had been chosen [12]. Reliability of the evaluation of this information in medical research articles has been shown to be good [13].

The theses were reviewed by one out of four assessors (PN, KS, MR and LR). Where the interpretation of the paper was ambiguous, it was appraised by other assessors and the conclusions reconciled in a group discussion.

The name of the student was then used to determine whether the thesis had resulted in a scientific publication indexed in the Medline, Scopus or Medic databases. The Medic database indexes were used to find scientific articles published in Finnish medical, dental and nursing journals. We searched for all articles that included the name of the student as an author and then browsed through these to find potential matches with the thesis. A thesis was considered to have been published if the title of one of these articles or the content of its abstract was consistent with the characteristics of the thesis. It was possible that some studies published in lower visibility journals not indexed in major databases were missed.

The journal impact factor as reported by the Journal Citation Reports of the ISI Web of Knowledge for the publication year was obtained from the journal that published the article. Journals not included in the ISI Web of Science system were assigned the impact factor value zero. These included all theses published in Finnish journals.

Citation counts for each published article were obtained from the Web of Science databases and the Scopus database in November 2006. The total number of citations received by each article counts the number of times other researchers have cited it in their subsequent publications during the period from the publication date to November 2006. Since the citation counts are affected by the follow-up time, which in this case ranged from 1 (published in 2005) to 6 (published in 2000) years, we used the average number of citations received per year to assess the utility of an article.

### Statistical methods

The data were analysed using SPSS for Windows 14.0 (SPSS Inc.; Chicago, Illinois, USA) software. Frequency distributions and cross-tabulations were used as the main tools for presentation and analysis. The distribution of the journal impact factor was visualised with box-plots.

## Results

### Characteristics of the theses

Of the 256 theses evaluated, 143 (55.9%) were clinical, 9 (3.5%) biomedical, 33 (12.9%) from a diagnostic department (Clinical Chemistry, Medical Microbiology, Forensic Medicine, Pathology or Diagnostic Radiology) or from the Department of Pharmacology and Toxicology, 22 (8.6%) from the Department of Public Health Science and General Practice, and 49 (19.1%) from the Institute of Dentistry.

In most cases the supervisor was a professor or adjunct professor (60.6%). The language was English in 71 cases (27.7%), Swedish in one, and the rest of the theses were written in Finnish.

A total of 119 theses (46.5%) reported quantitative research with samples mainly consisting of patient material, or in some cases experimental animals (n = 6). Quantitative research was linked with an extensive literature review or a report on the development of a laboratory procedure in 59 brief quantitative works (23.0%). Pure literature reviews accounted for 29 theses (11.3%). The number of reports of other type was 49 (19.1%), including five that were purely qualitative. Twenty reported extensive laboratory work, nine presented case reports or other descriptions and the rest were case reports accompanied by a literature review (n = 5), descriptions of laboratory procedure (n = 8) or analyses of qualitative material (n = 2).

Altogether 204 theses (81%) followed the structure of a scientific article, including an introduction, a description of the methods, a presentation of the results and a discussion section. In 142 cases (55.5%) the literature review made up more than half of the thesis. The technical quality was only satisfactory in 55 cases (21.5%), good in 124 (48.4%) and excellent in 63 (24.6%). Common shortcomings were variations in font size and style (30.9%), indistinct and unfinished figures and tables (17.6%), inconsistent line spacing (15.6%), indefinite margins (14.5%), inadequate use of references or a deficient list of references (12.1%), an unfinished table of contents (5.1%) and other defects (24.2%) such as the lack of an abstract, information lacking from the title page, or an otherwise disorganized report. A total of 14 theses (5.5%) were already available as printed publications and a further 52 (20.3%) were written according to the instructions of a specific scientific journal.

Statistical methods of at least some kind of were used in 178 (69.5%) theses that reported quantitative research. More than one fifth of those that had used statistical analyses included special methods such as survival analyses and multivariate models. Statistical figures and tests of statistical significance were presented in approximately half of the theses. Extensive description of the statistical methods used were presented in 69 theses (38.8%).

### Number and citation frequency of publications

A total of 61 theses (23.8%) resulted in a scientific publication, most of them were quantitative (62.3%). Those that were of excellent technical quality or originally written in English were more often published as scientific articles than those of poor technical quality or written in Finnish (Table 1). The distribution of publication by other characteristics is also shown in Table 1.

**Table 1:** Distribution of publications by characteristics of the theses.

	Publications			
		
Characteristics of the theses	No publications n (%)	Finnish n (%)	English n (%)	All n (%)
Field				
▪ Clinical medicine	104 (72.7)	6 (4.2)	33 (23.1)	143 (100)
▪ Biomedicine	4 (44.4)	0 (0)	5 (55.6)	9 (100)
▪ Diagnostics or pharmacology	23 (69.7)	0 (0)	10 (30.3)	33 (100)
▪ Public health science and general practice	20 (90.9)	1 (4.5)	1 (4.5)	22 (100)
▪ Dentistry	44 (89.8)	1 (2.0)	4 (8.2)	49 (100)
				
Type				
▪ Quantitative	81 (68.1)	3 (2.5)	35 (29.4)	119 (100)
▪ Brief quantitative	53 (89.8)	2 (3.4)	4 (6.8)	59 (100)
▪ Literature review	29 (100)	0 (0)	0 (0)	29 (100)
▪ Other	32 (65.3)	3 (6.1)	14 (28.6)	49 (100)
				
Supervisor				
▪ Professor	68 (68.7)	4 (4.0)	27 (27.3)	99 (100)
▪ Adjunct professor	46 (82.1)	1 (1.8)	9 (16.1)	56 (100)
▪ Other	67 (84.8)	2 (2.5)	10 (12.7)	79 (100)
▪ Unknown	14 (63.6)	1 (4.5)	7 (31.8)	22 (100)
				
Structure of scientific article				
▪ No	47 (95.9)	2 (4.1)	0 (0)	49 (100)
▪ Yes	148 (76.7)	6 (3.1)	39 (20.2)	193 (100)
▪ Printed publication	0 (0)	0 (0)	14 (100)	14 (100)
				
Language				
▪ Finnish or Swedish	169 (91.4)	8 (4.3)	8 (4.3)	185 (100)
▪ English	26 (36.6)	0 (0)	45 (63.4)	71 (100)
				
Technical quality				
▪ Poor	49 (89.1)	3 (5.5)	3 (5.5)	55 (100)
▪ Good	103 (83.1)	3 (2.4)	18 (14.5)	124 (100)
▪ Excellent	43 (68.3)	2 (3.2)	18 (28.6)	63 (100)
▪ Printed publication	0 (0)	0 (0)	14 (100)	14 (100)
				
Total	195 (76.2)	8 (3.1)	53 (20.7)	256

The student was the first author in 30 articles (49.2%), the second author in 21 (34.4%) and the third or later-mentioned author in 10 (16.4%). The supervisor was a co-author in 60 out of the 61 papers.

**Figure 1:**
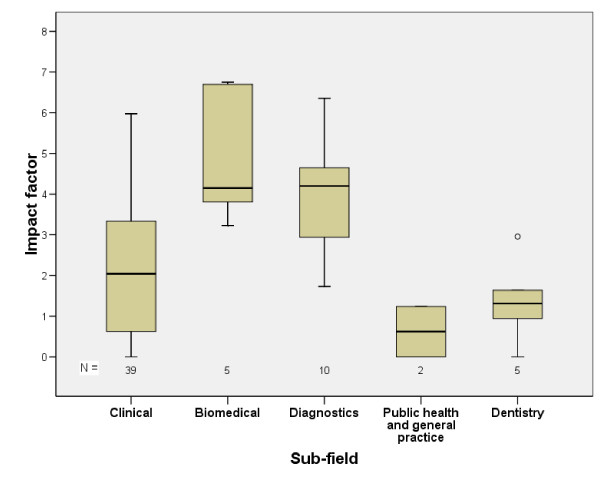
Distribution of impact factor by field. The horizontal line in the middle of the box is the median value for the journal impact factor and the lower and upper boundaries indicate the 25th and 75th percentiles. The largest and smallest observed values that are not outliers are also shown. Lines are drawn from the ends of the box to those values.

The distribution of impact factors by fields is shown in Figure 1. Theses in biomedical or diagnostic fields were more often published in highly cited journals than the others. Papers in purely clinical or dental fields were published in journals with lower impact factor than the others.

The median number of citations received per paper per year was 2.7, ranging from 0 to 14.7. There were four papers that were cited very often (more than 10 citations per year), three of which were quantitative and one of another type.

## Discussion

The present paper investigated the basic characteristics, publication pattern and utility of medical theses completed at the Faculty of Medicine, University of Oulu, Finland. The traditional format of reporting statistical analyses of medical data was most common, often combined with an extensive review of the literature or the development of a laboratory method. Most of these theses were supervised by university hospital clinicians. Shortcomings in the them were related to the technical quality and the description of the research methods. Most of the theses were not published, but the frequency of publishing the research findings in a scientific journal was higher for theses produced in more biomedical disciplines. The publications resulting from the theses received a moderate number of citations, which means that they were utilized quite well by other researchers.

For most physicians and dentists the thesis included in their basic medical and dental basic education is the only contact they have with the producing of new scientific information. The present analysis showed that regrettably often the student did not command the basics of scientific communication. These results are in agreement with other reports concerning the level of basic medical scientific education [8,14].

In 2001–2003, when these theses projects were carried out, the regulations regarding theses for the MD and DMD degree at the University of Oulu were somewhat cursory. They simply stated that the preparation of the thesis should include participation in data collection, preliminary observations and their processing in a research project and writing of a report on the research project. They also included requirements for the extent of the thesis and general instructions for writing it. No instructions were available for supervisors. This insufficiency of instructions for students and supervisors could partly explain some of the weaknesses in the theses.

Numerous books, articles and Internet sites offer recommendations and instructions for the students on how to write and organize a research paper or thesis. Medical and dental curricula often include introductions to the principles of scientific research, the retrieval of medical literature and data analysis, as in the University of Oulu [15]. Despite the existence of courses in medical informatics, guides and thesis regulations, many students do not understand the process of scientific writing [16]. Some medical schools have developed student-oriented courses and programmes to overcome the perceived difficulties and improve the quality of theses and promote their publication [7,8,17]. Our findings show that it is important to teach undergraduate students the full scientific publishing process, including the peer review process, the format for scientific articles and the necessary skills in word processing.

The weaknesses reported concerning the preparation of the theses may be associated with the lack of time [4]. Students' use of time in the undergraduate curriculum can be guided, and enough time should be reserved for this writing process. The courses of relevance to scientific education, such as scientific theory, information search and biostatistics, give tools for both completing the thesis and reading the scientific literature. These studies should be timetabled so that they support the actual work on the thesis in the best possible way.

The supervision of research is highly important, and the frequent lack of supervision is considered problematic [4,9]. The present analysis showed that the supervision was unevenly distributed within the clinics and departments: the proportion available in biomedical institutions being quite small, whereas a considerable amount of work was done in the clinical units. This is understandable, as thesis work usually takes place in the final stages of qualifying, when the students are studying clinical subjects. Furthermore, the variety in technical quality and content suggested that the supervisors were not fully aware of their responsibilities. Clear instructions and pedagogical education for supervisors could improve the quality of the theses and the fluency of the students' learning process. The supervisors and their departments and clinics should also be given proper acknowledgement for their work.

The strengthening of scientific education is a key component in developing competent physicians who will not only ask the right questions but also be able to apply current treatment methodologies [6]. The inclusion of research work in medical education can promote physicians' use of evidence-based medicine and their involvement in clinical trials. Lloyd et al. noted that participation in medical research as a student may be an important determinant of future involvement in clinical research [18]. In addition, experience from medical schools that have had student research programs suggests that these can and do encourage medical students to take an interest in research and possibly an academic career [19].

The diploma theses written by medical and dental students in Finland are not currently available on the Internet. In order to maximize the visibility and usage of student's work, medical and dental schools should make their reviewed diploma theses accessible to any potential users on the Internet. The full digital text of all theses can be deposited in the university's self-archive.

The percentage of diploma theses actually published (23.8%) is somewhat higher than the figures in two other European countries. A study in France showed that 17% of theses presented between 1993 and 1997 had resulted in publication by 1999 [5]. Frkovic et al. reported data on master theses defended at two Croatian medical schools [20]. They found that 14% of those prepared by medical students in 1990–1999 had subsequently been published in scientific journals indexed in Medline. The higher proportion of publications from the University of Oulu could be associated with the increase in medical publishing in Finland since the 1990s [21].

Publication in a peer-reviewed journal indicates that the content of the thesis is acceptable to the international scientific community. Our study showed that especially theses with a literature review were not published later in scientific journals. Writing a review of high scientific quality is a demanding process, and students may not have enough experience for this. The reasons for the thesis not resulting in publication may often lie elsewhere, however, and not in the quality or content of the project. The publication process is usually slow, and the student's most important goal at this stage may be graduation, the thesis being just a mandatory part of this. Also the publication process demands time and resources from the supervisor. On the other hand, publication is an additional merit for the supervisors, which should encourage them to aim at getting theses published.

One limitation of this study should be noted. It was performed in a local setting in one medical faculty in Finland – and each educational setting is unique. Nevertheless, in spite of the limited scope, our findings might be helpful when considering possible educational and training interventions in medical and dental schools which require the writing of a mandatory thesis, especially in Europe.

## Conclusion

Students and supervisors should be encouraged to aim at publishing the degree theses. The production of the thesis could mimic the scientific publishing process more closely than is currently the case. Requiring students to write their theses according to the guidelines of a few selected journals, improving the supervisor's engagement in the reporting and improving students' understanding of the peer review process would add a new dimension to the thesis process and provide additional opportunities for publication. The full digital text of the completed and reviewed thesis should be made visible and accessible in the institution's self-archive.

## Competing interests

The author(s) declare that they have no competing interests.

## Authors' contributions

PN and KS had the idea for the article. PN initiated the project, contributed to the data collection and statistical analysis and wrote the paper. KS, MR and LR contributed to the study design, data collection and writing of the paper. H-MT performed the bibliometric data collection and statistical analyses and contributed to the and writing of the paper. All the authors have read and approved the final manuscript.

## Pre-publication history

The pre-publication history for this paper can be accessed here:


